# Long-Term Follow-Up Examination of the Internal Jugular Vein After Vessel-Sparing Implantation of a Hickman Catheter or Port Catheter

**DOI:** 10.3389/fped.2019.00058

**Published:** 2019-03-13

**Authors:** Laura A. Ritz, Julia Ley-Zaporozhan, Dietrich von Schweinitz, Jochen Hubertus

**Affiliations:** ^1^Department of Pediatric Surgery, Dr. von Hauner Children's Hospital, Ludwig-Maximilian University, Munich, Germany; ^2^Department of Radiology, Medical Center of the Ludwig-Maximilian University, Munich, Germany

**Keywords:** vessel-sparing, Hickman-catheter, children, chemotherapy, parenteral nutrition, follow-up central catheter, patency after venous cut-down

## Abstract

**Introduction:** Both a Hickman catheter (HC) and port catheter (Port) can be inserted either percutaneously by the Seldinger technique or by surgical venous cut-down. Catheters are inserted with a vessel-sparing technique when they are placed in the internal jugular vein (IJV) by venous cut-down. Although this technique is common, data are sparse regarding the vessel's state at long-term follow-up. This study was aimed at determining the flow pattern and constitution of the IJV after vessel-sparing implantation of an HC or Port and comparing the outcomes to those of implantation with the Seldinger technique.

**Methods:** One hundred children (58 boys, 42 girls) between 33 days and 18 years of age who underwent a vessel-sparing implantation of an HC or Port in the IJV were prospectively included. All patients underwent surgical venous cut-down at a single institution. Patency and shape of the IJV were determined by ultrasound and categorized according to 2 possible outcomes: relevant alteration (including occlusion of the IJV) and no relevant alteration, with relevant alteration defined as changes that caused an altered flow pattern.

**Results:** Median age was 6 years at the time of operation, and the median indwelling time of catheters was 271 days. Twenty-two of our patients (22%) showed relevant alterations. These changes included high-grade stenosis or lesion in 13 patients (13%) and occlusion in 9 patients (9%). There were no operation-associated complications, such as pneumothorax, hematopericardium, or accidental puncture of the carotid artery. Statistical analysis did not reveal any specific parameter as a risk factor for relevant structural abnormalities.

**Discussion:** In a comparison of our data to the literature, venous cut-down showed an alteration rate of 26% and a patency rate of 85%, whereas the Seldinger technique was found to cause alteration in 15%, with a patency rate of 97% but a successful placement rate of only 90.3–91.6%.

**Conclusion:** The indication for long-term catheter placement may determine which method is preferable. A child who is likely to need more catheters in the future might benefit from the Seldinger technique, since there is a higher chance of long-term patency of the vessel. A patient undergoing chemotherapy might benefit more from the surgical venous cut-down with less placement-associated complications.

## Introduction

The Hickman® catheter (HC) and port catheter (Port-a-Cath®, Port) can be placed either percutaneously with the Seldinger technique or by surgical venous cut-down (1).

The catheter is tunneled subcutaneously and may last months or even years with good care and in the absence of catheter-associated complications such as infection, thrombosis, occlusion, secondary dislocation of the catheter, or material malfunction (2). A common indication for long-term catheter placement is chemotherapy in oncological patients (3). Correct placement of the catheter is essential, which is the main advantage of the venous cut-down method over the Seldinger technique. For the implantation, a midsized vein, such as the subclavian, cephalic, internal jugular vein (IJV), or external jugular vein, is needed (4). In order to maintain the vein's function, a vessel-sparing technique is used to place an HC or Port in the IJV (5).

Vascular changes after vessel-sparing surgery are rarely reviewed. Therefore, the aim of our study was to use ultrasound to determine vascular changes after vessel-sparing implantation of the catheter and to compare the results with data from the literature on the Seldinger technique. In addition, risk factors for venous alterations, including complications such as thrombosis, infection, or venous occlusion, were investigated.

## Materials and Methods

### Patients

We included all patients aged between 0 and 18 years, with unilateral and primary as well as secondary surgical catheter placement in the IJV, with a possibility to remove the catheter if no longer required or are causing problems. Exclusion criteria was death, at the time of the follow-up study.

Information about the study was sent to 339 patients and their parents in January 2017.

### Follow-Up

Most of the patients were treated at the Department of Pediatric Oncology and Hematology. We performed the ultrasound examination during the patient's regular check-up required for therapy at our clinic. The time interval between catheter removal and ultrasound examination ranged from the same day as catheter removal to 13 years afterward.

The study was approved by the ethics committee of the Ludwig-Maximilian University, Munich. Written informed consent was obtained from the patients or their guardians.

### Surgical Technique

To expose the IJV in the area of the cervical vertebrae 5/6 (C5, C6), a small transverse incision was made over the sternocleidomastoid muscle. After preparing the vein, 2 loops were placed to reversibly stop the blood flow. The catheter was tunneled subcutaneously from the anterior chest wall to the prepared vessel at the neck. A 6/0 polypropylene (Prolene, Ethicon, Somerville, NJ, USA) purse string suture was placed at the ventral side of the IJV, and the vessel was opened in the center along its long axis with a no. 11 blade scalpel (5). The correct position of the catheter's tip was determined with real-time fluoroscopy (6), and the catheter was secured with a purse string suture.

For the decannulation, the catheter was detached at the pectoral entry site and removed from the vessel while external pressure was applied at the cervix for 5–10 min.

### Ultrasound

We used a LOGIQ S8 (GE Healthcare, Buckinghamshire, UK) ultrasound system with a 2–8 MHz linear (9L) transducer. Ultrasound examination was performed by 2 experienced physicians. The patency and shape of the IJV was determined by ultrasound in M-Mode and flow characteristics were assessed by using the Doppler mode. If the vessel had no occlusion, it was carefully examined to detect any alteration, such as lesions, intraluminal mass, stenosis, dilatation, or presence of collateral veins. The contralateral IJV was used as an intra-individual control.

**Figure 1:**
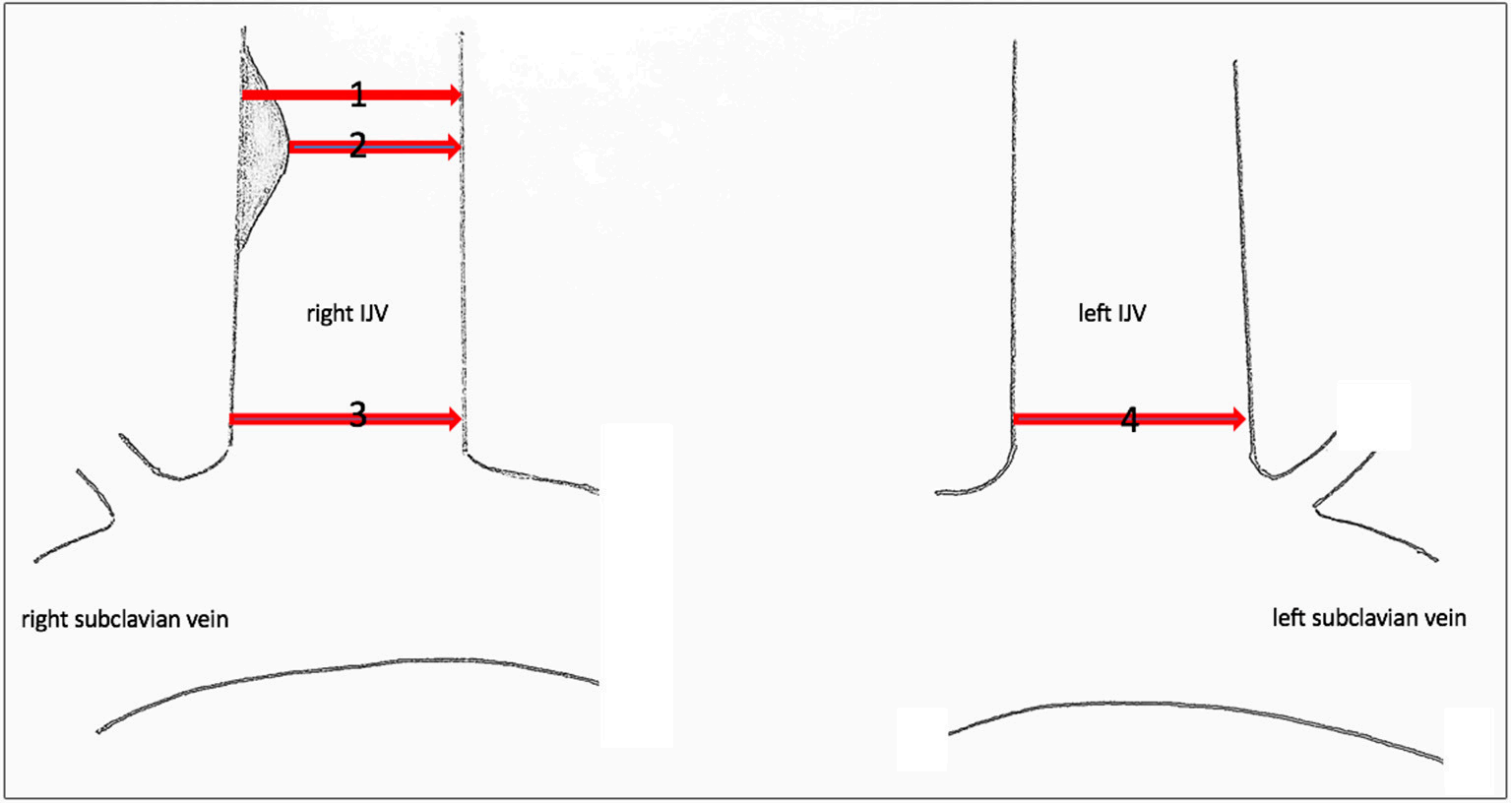
Sonographic measuring points of the IJV. Sonographic measuring points of the IJV to determine the grade of stenosis or dilatation. (1) Local diameter at the former catheter's entry site. (2) Residual diameter. The grade of stenosis caused by the endothelial lesion is calculated according to the ECST by the percentage diameter reduction. (3) Proximal dimension at the level of C7. The percentage of stenosis/dilatation is calculated relative to the diameter of the contralateral IJV, diameter 4, according to the NASCET.

Since no classification system exists for central venous stenosis, we used the European Carotid Surgery Trial (ECST) (7) and the North American Symptomatic Carotid Endarterectomy Trial (NASCET) (8) to categorize our findings. In both studies, the grade of stenosis of the internal carotid artery was classified. In the ECST, local stenosis was measured, and in the NASCET, the diameter of the vessel distal to the stenosis was measured (Figure 1) (9).

### Stenosis

The lumen dimensions of the IJV were measured by placing the ultrasound transducer horizontally at the level of C7. The percentage of stenosis was calculated in comparison to the diameter of the contralateral IJV. Since the diameter was measured proximal to the catheter's entry site, we applied the NASCET classification but used the contralateral vessel diameter at the same C7 level as a reference instead of the diameter before the catheter's entry location.

### Lesion

**Figure 2:**
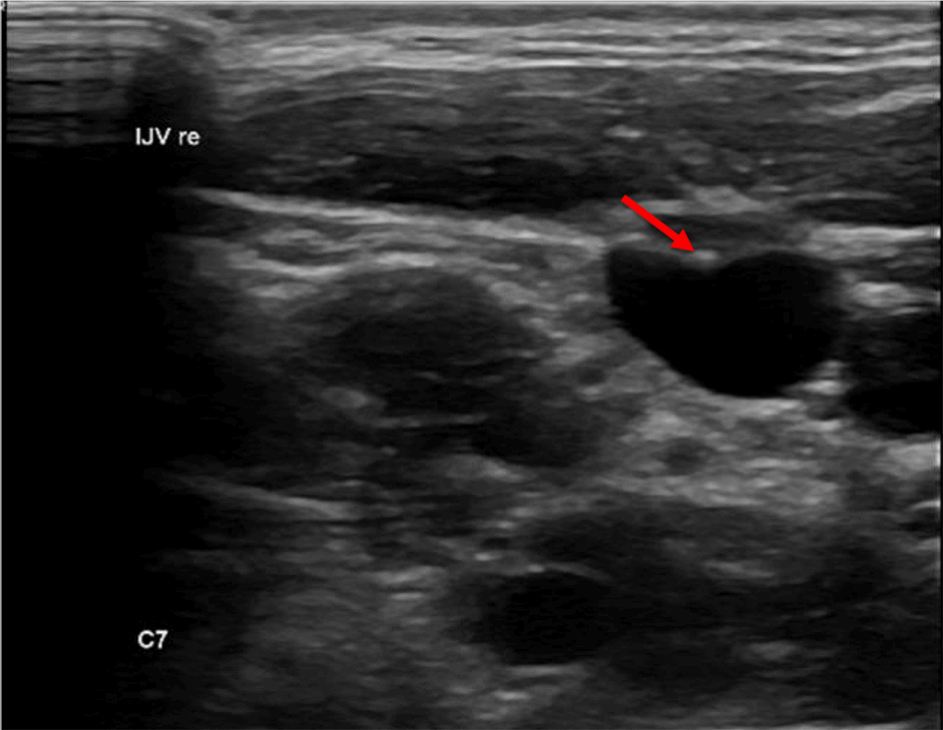
Endothelial lesion. Lesion at the catheter's former entry site of the vessel (red arrow).

**Figure 3:**
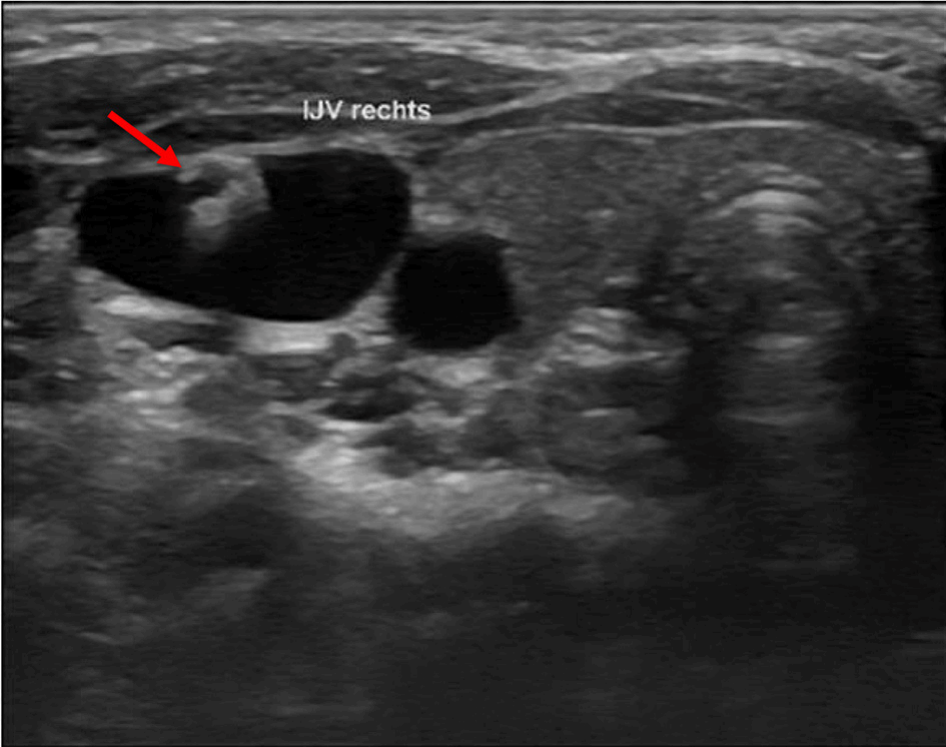
Intraluminal mass. The persistent thrombus (arrow) originating from the vessel entry site of the former catheter, forming a intraluminal polypous mass.

We defined a lesion as an alteration of the endothelium at the former catheter venous entry site (Figure 2). The lesion could be a visible lack or surplus of endothelium, caused by granulation tissue, a persistent thrombus or hypertrophic endothelium. We differentiated lesions that only appeared at the venous wall and those that compromised the venous lumen. We used the ESCT classification to grade the stenosis caused by the lesion. Therefore, the vessel's diameter was measured distal to the lesion and at the narrowest point of the lesion. The difference of the lumen dimensions determined the percentage of the stenosis according to the ESCT classification. An intraluminal mass emerging from the former catheter venous entry site was defined as a persistent thrombotic covering at the catheter's surface (Figure 3). It presented as polypous or as an intraluminal string.

The German Association of Ultrasound in Medicine (Deutsche Gesellschaft für Ultraschall in der Medizin [DEGUM]) offers an ultrasound classification of stenosis of the internal carotid artery (Table 1) (10), converting the NASCET and the ESCT. The classification includes Doppler sonographic findings, as an increase of the systolic peak flow or the reduction of the diastolic flow; the alias effect (estimated by a midgrade stenosis) as a sign of turbulence; or the confetti effect (high-grade stenosis) as a sign of perivascular vibration of the tissue due to highly increased flow just behind the area of stenosis [see Arning et al. (10) for the original classification]. Doppler sonographic findings are difficult to quantify in venous stenosis, so we focused our classification on the measured grade of stenosis, the alias effect (turbulences), and the presence or absence of collateral veins.

**Table 1:** Classification of internal carotid artery stenosis.

**DEGUM Grade**	**Plaque**	**Low**	**Mid**	**Mid-to-high**	**High**	**Very high**	**Maximum**	**Occlusion**
NASCET^a^ %	10	20–40	50	60	70	80	90	Occlusion
ESCT^b^ %	45	50–60	70	75	80	90	95	Occlusion

a *The NASCET measures the artery's diameter distal to the stenosis. For our study we used the classification for the diameter of the vein, analogically proximal to the former catheter entry site to calculate the extent of the venous stenosis. Accordingly the NASCET was used for venous dilatation*.

b *The ESCT is usually applied to classify the local stenosis of the carotid artery. We did the same with local stenosis of the vein, called lesion in our study*.

### Dilatation

A dilatation was classified according to the NASCET scheme. High-grade dilatation was defined as dilatation of 170% or more relative to the contralateral IJV.

**Figure 4:**
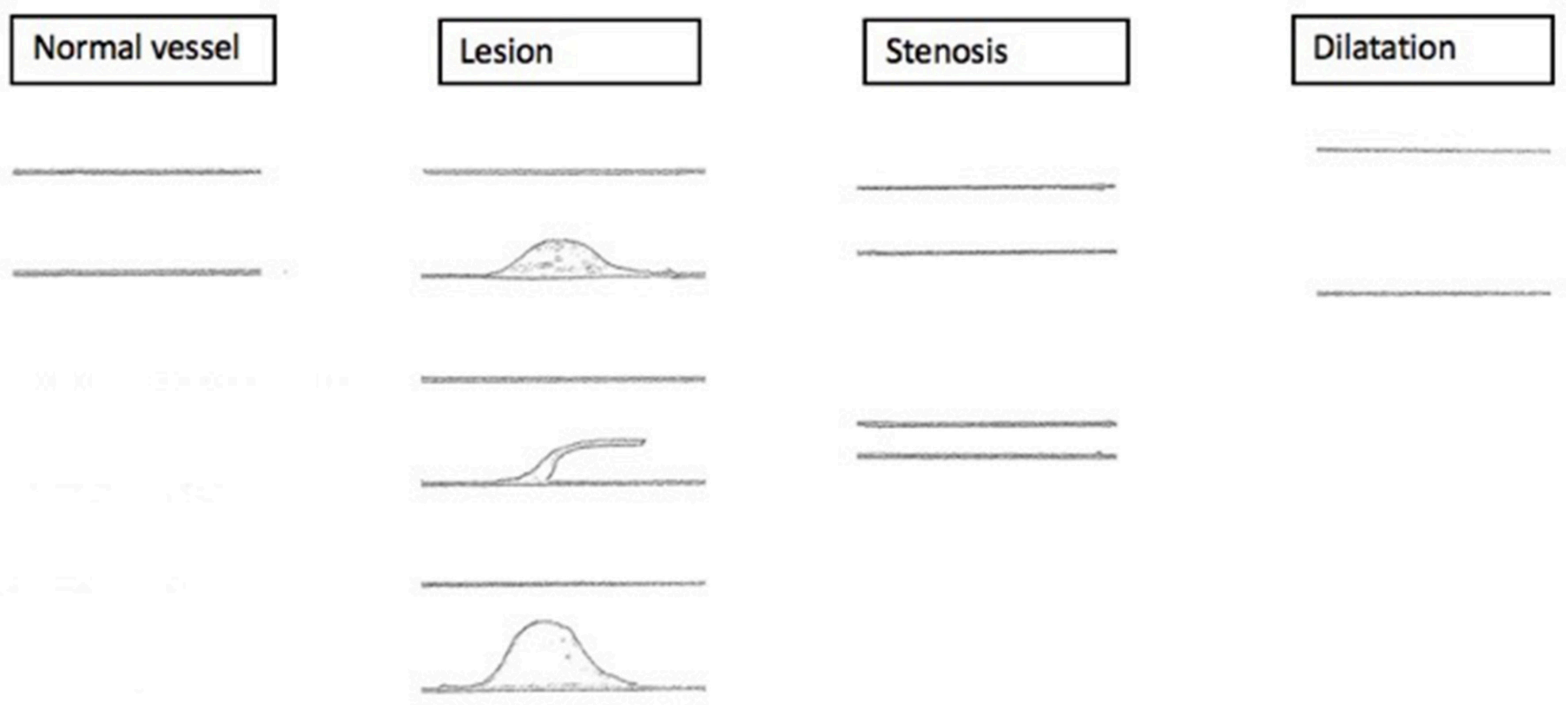
Sonographic findings. Schematized sonographic findings. (1) Lesion with mid-grade local stenosis. (2) Intraluminal mass, persistent thrombotic covering of the former catheter. (3) Lesion with high-grade local stenosis.

Lesion and stenosis (either local or proximal of the lesion) as well as lesion and dilatation can be simultaneously present (Figure 4).

Although the presence of intra-individual differences in vessel diameters is possible between the two sides, ultrasound prior to the operation is rare. Consequently, there were no baseline data for reference. Comparison to the contralateral side served only as a guideline.

### Occlusion

**Figure 5:**
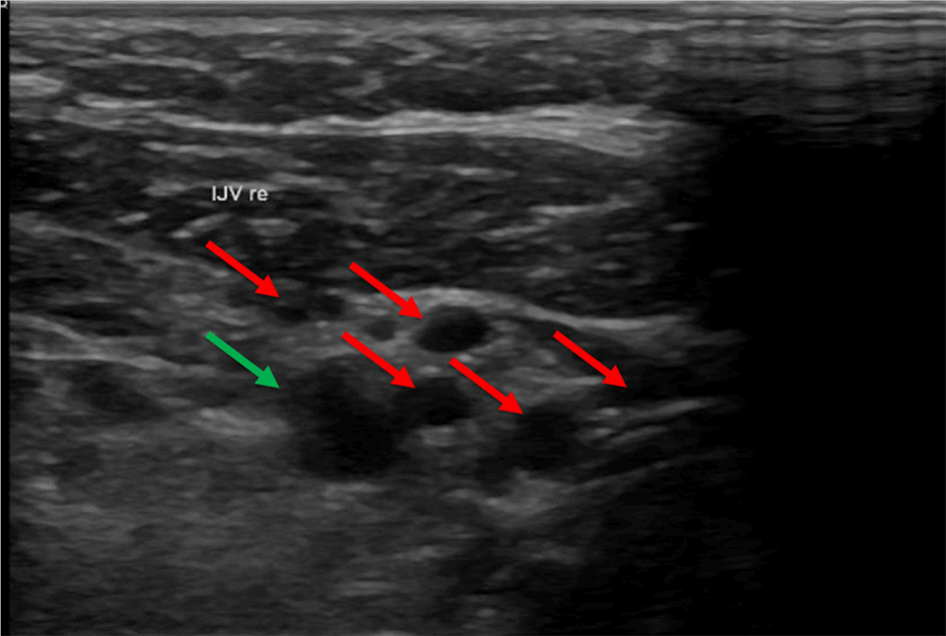
In this ultrasound image the carotid artery (green arrow) is surrounded by many collateral veins (red arrows) due to occlusion of the IVJ. In this ultrasound, the carotid artery is in the upper right part of the image. The small vessels surrounding the artery are collateral veins. The internal jugular vein (IJV) was absent.

In some cases, we found numerous collateral veins but no IJV. The IJV probably collapsed after removal of the catheter and degenerated over time. We referred to this circumstance as occlusion of the vessel (not to be confused with an occlusion of the catheter due to a thrombus) (Figure 5).

The outcome was classified according to two possible outcomes: relevant alteration and no relevant alteration. An alteration was classified as relevant when it caused an altered flow pattern or the existence of collateral veins, such as in the case of high-grade stenosis/lesion or more extensive damage (Table 2).

**Table 2:** Relevant and no relevant alterations.

**DEGUM-Grade**	**Plaque**	**Low**	**Mid**	**Mid-to-high**	**High**	**Very high**	**Maximum**	**Occlusion**
Stenosis %	10	20–40	50	60	**70**	**80**	**90**	**Occlusion**
Lesion %	45	50–60	70	75	**80**	**90**	**95**	**Occlusion**
Dilatation %	110	120–140	150	160	**170**	**180**	**190**	
Sonographic findings	None	None	None	None	**Turbulances**	**Turbulances**	**Turbulances**	**Collateral veins**

*Sonographic findings were classified as relevant when they caused an altered flow pattern or the existence of collateral veins. This table shows the grade of each alteration leading to those findings **(marked)**. All other findings were classified as not relevant*.

### Data Analysis

Sex, age at operation, indication for operation, side, operation time, catheter indwelling time, drugs administered via the catheter, catheter-associated thrombus or sepsis, patient's body mass index (BMI), coagulopathies, and surgeon's level of training were collected from medical records at ZPS SAP (SAP, Walldorf, Germany).

### Statistic Strategy

To identify a possible risk factor for the occurrence of relevant alterations, we performed multivariate linear regression with backward elimination followed by Pearson's chi-squared test using SPSS (IBM® SPSS Statistics, Version 25). As the independent variable we used patients' data from the analysis above. The significance level (a) was set to 5%.

### Comparison With the Literature

Since the Seldinger technique is not performed at our institution, we compared our findings with data from the literature.

## Results

There were 375 patients who underwent vessel-sparing implantation of an HC or Port between July 2004 and April 2017 at our institution. Thirty-six patients had to be excluded (20 patients were lost to follow-up, and 16 patients were deceased). During the period March 2017 to December 2017, we examined 103 patients. Three had to be excluded from the study: 2 because of incomplete patient data and 1 because of a complication at decannulation, in which the IJV had to be ligated because of secondary hemorrhage. In the remaining study population of 100 children, the patient's age at the time of the operation ranged between 33 days and 18 years, with a mean age of 6 years. Seventeen patients (17%) were younger than 1 year and 83 were older (83%). Fifty-eight patients (58%) were male and 42 (42%) were female. Eighty-five (85%) catheters were implanted in the right IJV and 15 (15%) in the left IJV. In 93 cases (93%) the HC or Port was used for chemotherapy or stem cell transplantation, and in 7 cases (7%), it was used for parenteral nutrition (PEN).

**Figure 6:**
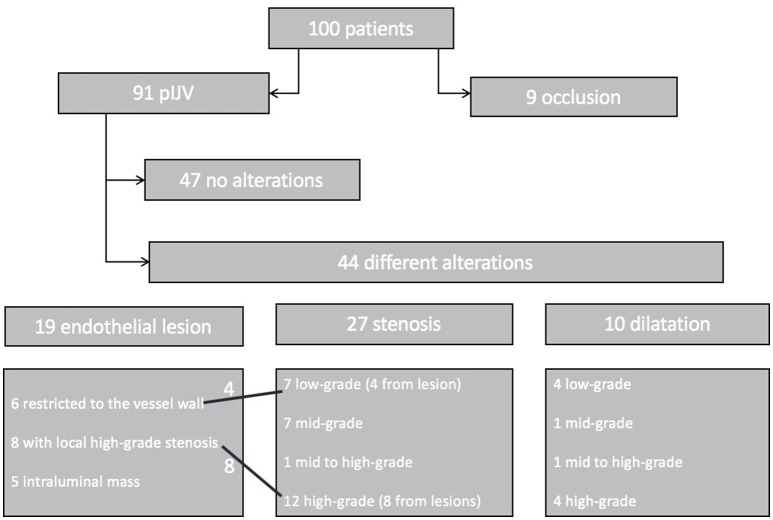
Patient flowchart. One hundred patients were included in the follow-up. Nine showed an occlusion of the IJV. The remaining 91 patients were subjected to thorough sonographic examination. Forty-seven patients had a normal vessel, 44 patients showed alterations such as endothelial lesions (19 patients), stenosis (27 patients), or dilatation (10 patients). Four of the endothelial lesions caused low-grade stenosis, and 8 caused high-grade stenosis. Some of the patients had two findings (e.g., lesion with local stenosis and a proximal stenosis of the vessel).

Nine patients (9%) presented with occlusion of the IJV after decannulation. Ninety-one patients (91%) presented with patent IJV (pIJV): 47 patients (47% of all patients) showed no alterations at all, and 44 patients (44%) had vessel alterations. Among the 44 cases with alterations, 13 (13% of the entire patient population) were classified as relevant (alterations causing an altered flow pattern, such as in the case of high-grade stenosis or lesion). In addition, the 9 patients with occlusion were classified as having relevant alterations (Figure 6).

In 19% of the patients, the alterations were local endothelial lesions: in 6 patients the lesion was restricted to the vessel wall and did not compromise the lumen: in 8 patients the lesion caused a high-grade or higher stenosis based on our modified DEGUM classification; and in 5 patients we detected a hyperechogenic intraluminal mass. The flow pattern was not altered in these patients.

In 27 patients (27%) we found stenosis of the IJV: 7 patients showed low-grade stenosis; 7 patients mid-grade stenosis; 1 patient mid- to high-grade stenosis; and 12 patients high-grade stenosis. In the patients with high-grade stenosis, collateral veins were more numerous. High-grade and higher stenosis were associated with an alteration of the Doppler sonographic flow pattern, such as turbulence.

Dilatation of the IJV was detected in 10 patients (10%): 4 patients had low-grade dilatation (<145% the lumen of the contralateral side); 1 patient, midgrade dilatation; 1 patient, mid- to high-grade dilatation; and 4 patients had high-grade dilatation. None of these cases were accompanied by a relevant modified Doppler ultrasound flow pattern.

Eight patients (8%) had a gene mutation or metabolic condition that could lead to an increased risk of thrombosis. Two patients (2%) had a lipoprotein a level over 30 mg/dL (patient 1, 148 mg/dL; patient 2, 344 mg/dL). Because of the increased risk of thrombosis, both patients were on anticoagulative therapy (Enoxaparin-Sodium, anti-Xa level 0.6–0.8). While the HC was indwelling, neither patient experienced catheter-associated thrombosis. At the follow-up examination, patient 1 showed an occlusion of the IJV, while patient 2 had no alteration of the IJV.

Two patients (2%) had a homozygous mutation of the Factor-V-Leiden (*FVL*) gene. At the follow-up, both showed a pIJV, and one had a relevant alteration. Four patients (4%) had a mutation of the methylenetetrahydrofolate reductase gene (*MTHFR*), which leads to an increased level of homocysteine. This mutation was homozygous in 2 cases and heterozygous in the 2 other cases. One patient (1%) had both a homozygous *MTHFR* mutation and a homozygous *FVL* mutation as well as a tumor-associated thrombosis. This patient showed a pIJV with a lesion that caused high-grade local stenosis. Both patients with the heterozygous *MTHFR* mutation had a catheter-associated thrombus and underwent anticoagulative therapy with Enoxaparin-Sodium for 3 months (anti-Xa level 0.6–0.8). At the ultrasound examination, 1 patient presented with high-grade stenosis, while the other had no alteration of the IJV.

Two patients (2%) had a tumor-associated thrombus in their central veins and had anticoagulative therapy with Enoxaparin-Sodium (anti-Xa level 0.4–0.6) for 3 months. One patient had high-grade stenosis, while the other patient had mid-grade dilatation at the follow-up examination.

While the catheter was indwelling, 8 patients (8%) developed catheter-associated sepsis. One patient (1%) developed a catheter-associated thrombosis and was treated with Enoxaparin-Sodium (anti-Xa level 0.6–0.8). On our ultrasound examination, this patient presented with an endothelial lesion. One patient also had a homozygous *MTHFR* mutation, but Doppler-sonographic changes were not observed. Four patients (4%) showed stenosis at the follow-up examination: 2 patients (2%) had a mid-grade stenosis, one of them had the HC for PEN; 1 patient (1%) had high-grade stenosis; and 1 patient (1%) had an occlusion of the IJV. One patient (1%) had high-grade dilatation found in the follow-up scan. One patient (1%) did not show any alterations at all.

Two patients (2%) experienced hypertension and were given antihypertensive drugs. They showed no alterations in the follow-up examination.

Three patients (3%) were born premature (34 + 4, 24 + 6, 25 + 2 weeks of gestation). One patient (1%) showed a relevant alteration.

One patient (1%) had an HC implanted in the same IJV twice, and this patient showed a persistent intraluminal thrombus.

The exact operation time was verifiable for 70 patients. Operations ranged from 20 to 115 min, with a median operation time of 56 min. In 23 patients (23%) the operation time was longer than 60 min, and 8 of these patients showed a relevant finding.

The catheter indwelling time ranged from 25 to 1,376 days, with a median time of 271 days. In 12 patients (12%), the catheter was in use longer than 1 year, but only 5 of these patients (5%) exhibited a relevant alteration of the vessel.

Twelve patients (12%) had a normal weight (BMI 18–24), 83 (83%) were underweight (BMI < 18), and 5 patients (5%) were obese (BMI >25) at the time of operation.

In 76 cases (76%) a single- or double-lumen HC (C.R. Bard Inc., Karlsruhe, Germany) of 6 to 9 French (Fr) was implanted. In 18 cases (18%) a 3-lumen with a 10-Fr catheter was placed. Only 6 patients received a Port-a-Cath (Bard Access Systems, Inc.; Salt Lake City, UT). All patients with a Port-a-Cath showed an alteration of the IJV, and for 2 of them, the alterations were classified as relevant.

The surgeon's level of training ranged from resident physician (year 2 to 6) with assisting attending physician to head of department. Analysis of medical records did not include the operating surgeon in 8 cases (8%) because the operation occurred prior to the installation of the electronic chart. In 50 cases (50%) the surgery was performed by a resident physician, and in 42 cases (42%) by a board-certified pediatric surgeon. Twenty-two patients (22%) showed a relevant alteration of the IJV (13 high-grade stenosis, 9 occlusion). Of these patients, 9 were operated on by a resident physician, 11 by a board-certified pediatric surgeon, and 2 by a surgeon with an unknown level of training. The decision of whether a resident or board-certified physician did the operation was made according to the expected level of difficulty and the age of the patient (Table 3).

**Table 3:** Patient data and Doppler sonographic findings.

**Parameter**	**Number of patients**	**No relevant alterations**	**Relevant alterations**	**Statistical correlation with relevant alteration**
All patients	100	78	22	
Male	58	44	14	0.67
Female	42	34	8	0.67
Right	85	87	18	0.18
Left	15	11	4	0.18
Age < 1 year	17	12	5	0.73
Age > 1 year	83	6	17	0.73
Patent IJV	91	78	13	
Occlusion of IJV	9	0	9	
Lesion/ persistent thrombus (at the catheters entry site)	19	11	8	
Stenosis of the IJV	27	15	12	
Dilatation of the IJV	10	10	0	
Increased risk of thrombosis	8	5	3	(0.44)
Lipoprotein a > 30 mg/dl	2	1	1	(0.43)
Homozygote FVL-mutation	2	1	1	(0.43)
MTHFR- mutation	5	3	2	(0.70)
Homozygote	2	1	1	
+ homozygote FVL +tumor-ass. Thrombus	1	0	1	
Heterozygote	3	2	1	
Tumor-associated thrombus	2	0	2	(0.02)
Catheter-associated thrombus during catheter's indwelling	4	3	1	(0.96)
Catheter-associated sepsis	8	6	2	(0.05)
+ thrombus	1	1	0	
Arterial hypertension	2	1	1	(0.43)
Premature birth	3	2	1	(0.68)
Secondary placement of HC/Port at the same IJV	1	1	0	(0.28)
Operation time < 60 min	47	36	11	0.55
Operation time > 60 min	23	15	8	0.55
Catheter's indwelling time < 365 days	85	64	21	0.27
Catheter's indwelling time > 365 days	12	7	5	0.27
Level of surgeon training = resident physician	50	41	9	0.54
Level of surgeon training ≥ board-certified pediatric surgeon	42	31	11	0.54
BMI 18–24 (normal weight)	12	9	3	0.39
BMI < 18 (underweight)	83	61	22	0.39
BMI >24 (obesity)	5	5	0	0.39
HC single- or double-lumen	76	57	19	0.58
HC tripple-lumen	18	17	1	0.58
Port-a-Cath	6	4	2	0.58
PEN	7	4	3	(0.29)

*All patient data were categorized according to the two outcomes: relevant and no relevant alteration. Relevant alterations were defined as high-grade and more extensive stenosis or occlusion of the IJV, which caused a Doppler sonographic alteration of the blood-flow pattern. The statistical column shows the correlation between the parameter and relevant alteration. Some results are given in parentheses due to the small number of patients*.

Statistical analysis using multivariate linear regression with backward elimination suggested a correlation between pIJV and operation-time (*p* = 0.016, standard deviation = 0.012, regression coefficient = −0.029); however, this result could not be confirmed in further analysis using univariate linear regression (*p* = 0.944, standard deviation = 0.001, regression coefficient = 6.594E-5). Sepsis and relevant alterations appeared to be correlated in Pearson's chi-squared test (*p* = 0.05), but this finding should be interpreted carefully because only 8 patients experienced sepsis. Age, infection, thrombosis, prematurity, and indwelling time were not significantly correlated with relevant alterations of the vessel.

The time interval between catheter removal and follow-up examination ranged from the same day as catheter removal to 13 years afterward. Lesions with an intraluminal mass (found in 5 patients) were only seen in the 1-year follow-up period and showed statistical correlation. Occlusion (9 patients) was detected in 6 cases after 5 years or longer (54%), in 2 patients between 1 and 5 years (18%) and only in 1 patient (9%) before 1 year. All other findings occurred in every time interval after catheter removal (Table 4).

**Table 4:** Alterations at the follow-up examination.

**Follow-up period**		**<1 year**	**1-5 years**	**> 5 years**	**Sum**
**STATISTICAL CORRELATION** ***P*** **=**					
Not relevant		16 *p* = 0.68	28 *p* = 0.69	34 *p* = 0.97	78
Relevant		6 *p* = 0.68	6 *p* = 0.69	10 *p* = 0.97	22
Lesion		11 *p* = 0.18	5 *p* = 0.83	3 *p* = 0.16	19
	Restricted to vessel‘s wall	2 *p* = 0.31	2 *p* = 0.62	2 *p* = 0.69	6
	Causing high-grade stenosis	4 *p* = 0.39	3 *p* = 0.34	1 *p* = 0.09	8
	With intraluminal mass	5 ***p*** **= 0.00**	0 *p* = 0.09	0 *p* = 0.06	5
Stenosis		9 *p* = 0.16	11 *p* = 0.93	7 *p* = 0.17	27
	Not relevant	4 *p* = 0.09	7 *p* = 0.49	4 *p* = 0.35	15
	Relevant	1 *p* = 0.09	1 *p* = 0.49	2 *p* = 0.35	4
	Relevant stenosis caused by lesion	4 *p* = 0.39	3 *p* = 0.34	1 *p* = 0.09	8
Dilatation		2 *p* = 0.32	4 *p* = 0.81	4 *p* = 0.32	10
Occlusion		1 *p* = 0.34	2 *p* = 0.43	6 *p* = 0.12	9
No of patients		22	34	44	

*The alterations shown at the follow-up examination according to the time interval after catheter removal. Intraluminal masses were exclusive to the 1-year period, and occlusion mostly occurred after 5 years. All other lesions occurred at any time. Statistical correlation was added*.

## Discussion

Chemotherapy, PEN, and stem cell transplantation are common indications for a long-term venous catheter placement in pediatric patients. Safe placement of the catheter in the central vein is vital because a malposition can cause severe side effects or death. Our study showed successful implantation of a catheter in the intended vessel in 100% of patients, using the open venous cut-down technique compared to 90.3% (11) or 91.6% (12) using the Seldinger technique in children.

The mean operative time performing a venous cut-down in this study was 56 min (range 20–115 min). Although the operative time was significantly lower when using percutaneous access [range 8–80 min, with a mean operative time of 19 min; (12)], the occurrence of direct placement-associated complications such as pneumothorax [subclavian vein access; (13)], hematopericardium [deep insertion of the guidewire (12)], venous dissection (11) or accidental carotid artery catheterization (14) has only been reported with the Seldinger technique (15). None of the mentioned complications occurred in our study population.

In a study of 23 neonates, Kim et al. (16) described vascular alterations after venous cut-down in 6 of the cases (26%). The alterations included venous occlusion in 1 patient (4.4%) and an altered Doppler sonographic flow pattern due to intravascular masses or changes in the venous wall flexibility in 5 cases (21.6%). Risk factors for the occurrence of vascular alterations were low body weight, low gestational age, and a longer duration of catheter use (16). Our findings were similar to the results from that study. Forty-four of our patients (44%) had venous lesions or persistent intraluminal thrombus, stenosis, or dilatation, some of the patients with more than one condition. In 9 patients (9%) occlusion of the IJV was found. Altogether, findings were classified as relevant in only 22 patients (22%) due to effects on the blood-flow pattern. Our study population differed from the population studied by Kim et al. (16), who examined only neonates. In our infant (< 1-year-old) subgroup, 5 of 17 patients (29%) had relevant findings and 3 (17.6%) had occlusion. However, there was no statistical correlation between relevant findings or occlusion and the age, longer catheter indwelling time, or low gestational age.

Similar results were documented by Willetts et al. (17). They examined 79 patients who underwent venous cut-down prior to a second implantation of a long-term catheter. In Doppler sonography, 70 IJVs (88.6%) appeared patent with no relevant alteration, 3 veins (3.8%) showed stenosis, and 6 veins (7.6%) were obliterated. In the following surgical exploration, 7 of the ultrasonographically patent veins (10.6%) showed an obliterated IJV with enlarged collateral veins, which could be responsible for the false-positive sonographic results. The proven patency was 74.6% (59 patients).

After surgical venous cut-down, approximately one-fourth of the veins show signs of alteration. This outcome appears to be the main disadvantage of the surgical venous cut-down over the Seldinger technique, in which the vein is only punctured rather than opened and sutured. The patency of the IJV at follow-up examination after ultrasound-guided percutaneous HC insertion was investigated by Wragg et al. (18). They recorded a patent vein in 97 cases (97%) right after the removal of the catheter. The study population was very similar to ours, with 100 patients and a mean age of 6 years. Twelve of their patients (12%) showed reduced flow, which was caused by intraluminal thrombosis or narrowing of the vessel. Therefore, even with the Seldinger technique, only 85 patients (85%) did not show any signs of alteration (18).

Since intraluminal masses are seen exclusively in the first year after catheter removal, it seems that this alteration changes after time. A possible explanation is a transient thrombotic covering of the catheter's surface. A larger study population would be needed to make a definite conclusion.

To determine the grade of stenosis we modified the existent classification of stenosis of the carotid artery, ESCT and NASCET. Since the wall structure in veins is less persistent because of the tunica media, which contains smooth muscle tissue, the measurements are not as exact and reproducible as in arteries. Because of the reduced fraction of muscle cells, the venous lumen differs according to the pressure applied from the outside (ultrasound transducer) (6) or the inside (valsalva maneuver) as well as the position of the patient's head.

Performing multivariate linear regression with backward elimination, we found an inverse statistical correlation of pIJV and OR-time. In the univariate linear regression, this correlation could not be confirmed. Pearson's chi-squared test revealed a correlation between sepsis and relevant alteration, but this finding should be interpreted carefully because only 8 patients experienced sepsis. Age, infection, thrombosis, prematurity, and indwelling time were not significantly correlated with relevant alteration or specifically with pIJV, which demonstrates a lack of predictors or risk factors for the occurrence of a relevant alteration.

## Conclusion

This study showed that the longer operation time of vessel-sparing venous cut-down is justified by successful and correct placement of the catheter in the intended vessel in 100% of patients. Since correct placement is indispensable for most indications for HC or Port and allows immediate use of the catheter, this factor is the main advantage of the venous cut-down over the Seldinger technique, which has successful placement in only 90–92% of cases. Even if 22% of our patients had relevant structural alterations of the IJV at long-term follow-up, no specific parameter increased the risk for structural alterations of the vessel. That finding demonstrates that the vessel-sparing venous cut-down technique has the same prospects for success, despite risk factors. The wide time interval of the follow-up examination after catheter removal helps to reveal the dynamics of the alteration. According to our data, intraluminal masses are only temporary alterations, whereas every other alteration seems to be permanent.

In a comparison of our data with published data on the Seldinger technique and venous cut-down, the Seldinger technique seems to be the least traumatic technique. It had relevant alteration in only 15% of cases and occlusion in 3% compared to venous-cut down in our study, with relevant alteration in 22% and occlusion in 9%, and bibliographic data on venous cut-down, with relevant alteration in 29% and occlusion in 25%. Even if the difference between relevant alterations in our data and the published data on Seldinger technique is small, it is worth considering when choosing the best technique. If a child is facing chemotherapy and is likely to need a long-term catheter only for a specific time, the benefit of a catheter safely placed by sight that is ready to use immediately might outweigh the alternative. Whereas a patient who is likely to need more than one catheter in the future (e.g., short-bowel-syndrome) might benefit from an approach with a lower risk of permanent changes of the vessel, which would be enabled by the Seldinger technique.

## Ethics Statement

Study was approved by the ethical committee of the University of Munich, Number 661-16 and written consent was obtained of every participant.

## Author Contributions

JH and LR: study design, data collection, and preparation of the manuscript. JL-Z: data collection and reviewed the manuscript, overlooking of the sonographic follow-up. DvS: study design and reviewed the manuscript.

### Conflict of Interest Statement

The authors declare that the research was conducted in the absence of any commercial or financial relationships that could be construed as a potential conflict of interest.
